# A Giant Solitary Fibrous Tumour of the Pleura

**DOI:** 10.7759/cureus.24062

**Published:** 2022-04-12

**Authors:** Younus Qamar, Maryam Gulzar, Hannah Yonis, Haytham Sabry, Tariq Minhas

**Affiliations:** 1 Department of Cardiothoracic Surgery, The Essex Cardiothoracic Centre, Basildon & Thurrock University Hospital, Mid and South Essex NHS Foundation Trust, Basildon, GBR; 2 Department of Cardiothoracic Surgery, Liverpool Heart and Chest Hospital NHS Foundation Trust, Liverpool, GBR

**Keywords:** paraneoplastic syndromes, right posterolateral thoracotomy, pleural mass, pleural neoplasm, hypertrophic pulmonary osteoarthropathy, benign lesion, surgical resection, pleural tumour, pleural fibroma, solitary fibrous pleural tumour

## Abstract

A solitary fibrous tumour of the pleura (SFTP) is a rare pathology, frequently benign in nature, and is usually diagnosed incidentally on imaging. We herein describe the case of a previously fit and well, 35-year-old Caucasian lady, who presented to us with a history of progressively worsening shortness of breath. Her chest X-ray showed a near-complete opacification of the right hemithorax, with displacement of the mediastinum towards the left. This study was supplemented by a computed tomography (CT), which demonstrated a well-circumscribed, non-homogenous mass, occupying the entirety of the right hemithorax. A large, smooth, encapsulated tumour was surgically resected via a posterolateral thoracotomy, measuring approximately 23.1 cm x 21.0 cm x 11.5 cm and weighing 3640 grams. Histopathology confirmed the diagnosis of a benign SFTP with an intermediate malignant potential. At six months, a follow-up CT scan demonstrated no evidence of disease recurrence.

## Introduction

Solitary fibrous tumours of the pleura (SFTP) are rare, mesenchymal cell tumours, accounting for <5% of all pleural tumours [1]. They are usually benign in nature, and frequently asymptomatic [2]. In approximately 80% of cases, the tumour originates from the visceral pleura [3]. Clinically, symptoms may include atypical chest pain or tightness, dyspnoea, haemoptysis, and fever [2,4]. Surgical resection of the tumour is the treatment of choice with an excellent prognosis. Surveillance with serial computed tomography (CT) scans at 6-monthly intervals is recommended to monitor for disease recurrence [1,2,4]. Herein, we present a case of a 35-year-old Caucasian lady, who presented with a six-week history of worsening dyspnoea, and was found to have a large, non-homogenous mass, encompassing the entirety of the right hemithorax and compressing the adjacent lung parenchyma.

## Case presentation

A previously fit and well, 35-year-old Caucasian lady presented to her general practitioner (GP) with a six-week history of progressively worsening shortness of breath. She had no significant past medical or surgical history. She denied any history of tobacco or asbestos exposure and had no family history of lung cancer or pulmonary disease. Her blood pressure was 138/74 mmHg, pulse rate was 72 beats/min and respiratory rate was 24 breaths/min. She was afebrile and her oxygen saturation was 95% on room air. Clinical examination of the chest revealed decreased breath sounds over the right hemithorax and digital clubbing. Blood investigations were unremarkable, except for a mildly elevated C-reactive protein (CRP) of 11 mg/ml. The preliminary diagnosis was a right-sided pleural effusion.

**Figure 1:**
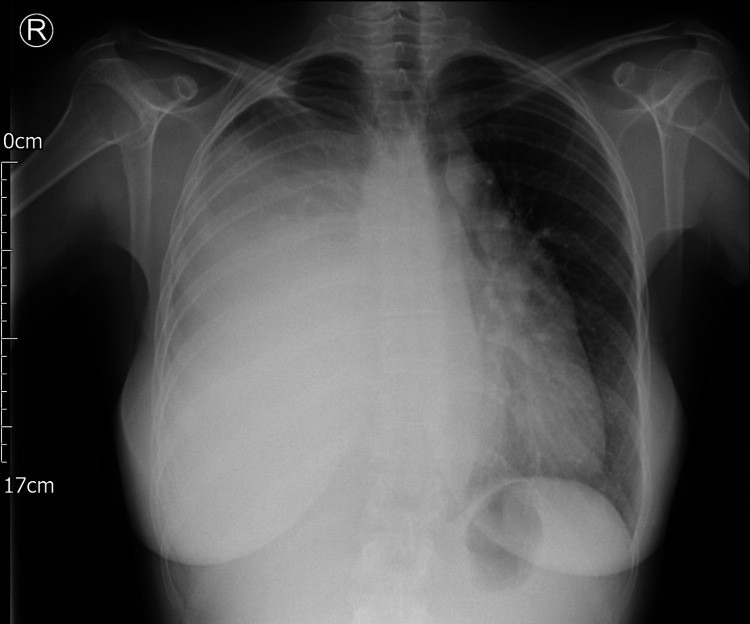
A posteroanterior (PA) chest X-ray showing a near-complete opacification of the right hemithorax with displacement of the mediastinum towards the left.

To confirm the diagnosis and help guide further management, a chest X-ray was requested. It showed a near-complete opacification of the right hemithorax with displacement of the mediastinum towards the left (Figure 1).

**Figure 2:**
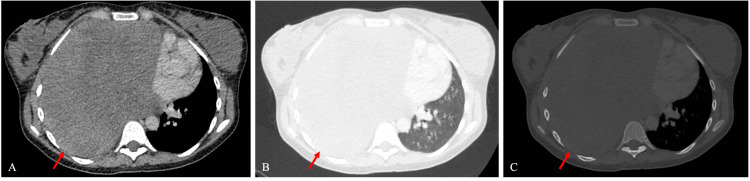
A contrast-enhanced computed tomography (CT) scan showing a large, low-density mass in the right hemithorax with moderate mediastinal shift towards the left (red arrow). There was no obvious destruction of adjacent bones. Panel A shows the mediastinal window, demonstrating the chest wall and pleura. Panel B shows the lung window, demonstrating the lung parenchyma in detail, including the pulmonary vasculature. Panel C shows the bone window.

A contrast-enhanced computed tomography (CT) scan of the chest was performed. It demonstrated a large, low-density area, encompassing the entirety of the right hemithorax with moderate mediastinal shift towards the left, and compressing the liver (Figure 2). The density was not in keeping with simple fluid, i.e., not typical for a pleural effusion, thus shifting the likely diagnosis towards a haematoma or a mass. It was a well-circumscribed, non-homogenous mass, adherent to the pleura. Additionally, the CT scan revealed an 11 mm left axillary lymph node and an enlarged liver, measuring 20 cm in the craniocaudal dimension.

**Figure 3:**
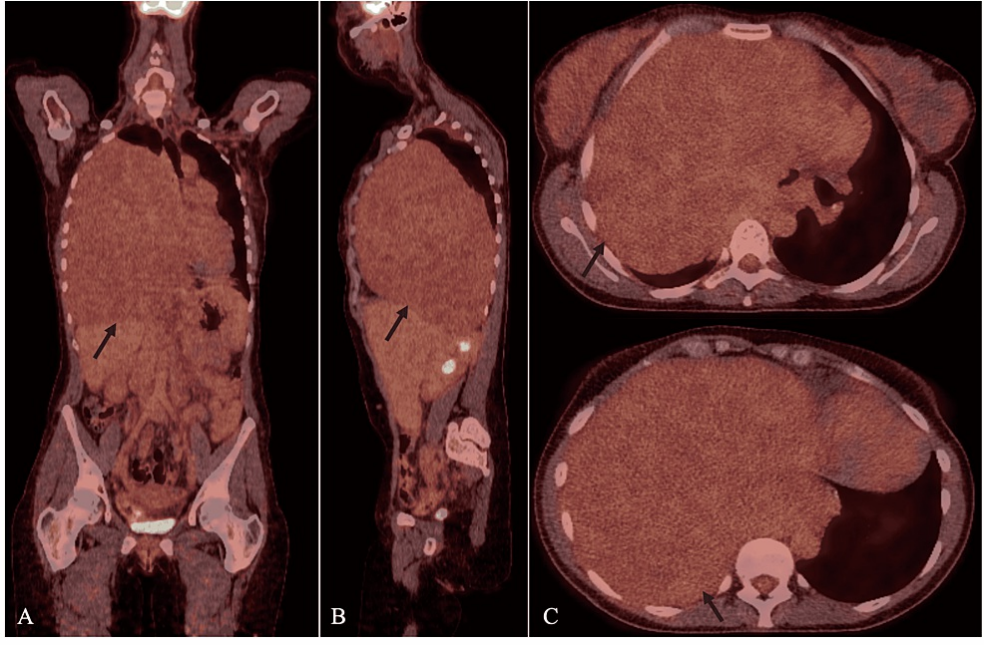
Positron-emission tomography (PET) images demonstrating a large mass, measuring approximately 25 cm x 15 cm, occupying the entire right hemithorax (black arrow). The maximum standardised uptake value (SUV max) was 2.5, similar to the rest of the mediastinal uptake. No fluorodeoxyglucose (FDG)-avid mediastinal or hilar lymph nodes were present. Panel A shows a coronal view. Panel B shows a sagittal view. Panel C shows two slices in axial views.

Subsequently, an ultrasound-guided biopsy of the mass was performed; however, the histopathology was inconclusive. This case was discussed in a multi-disciplinary team (MDT) meeting, where the consensus was to proceed with surgical excision of the tumour, preceded by a positron-emission tomography (PET)-CT scan. The PET-CT scan confirmed the presence of a large mass, measuring approximately 25 cm x 15 cm, occupying the entire right hemithorax (Figure 3). The maximum standardised uptake value (SUV max) was 2.5 (signifying low-metabolic activity), similar to the rest of the mediastinal uptake. Furthermore, there were no suspicious mediastinal or hilar lymph nodes.

**Figure 4:**
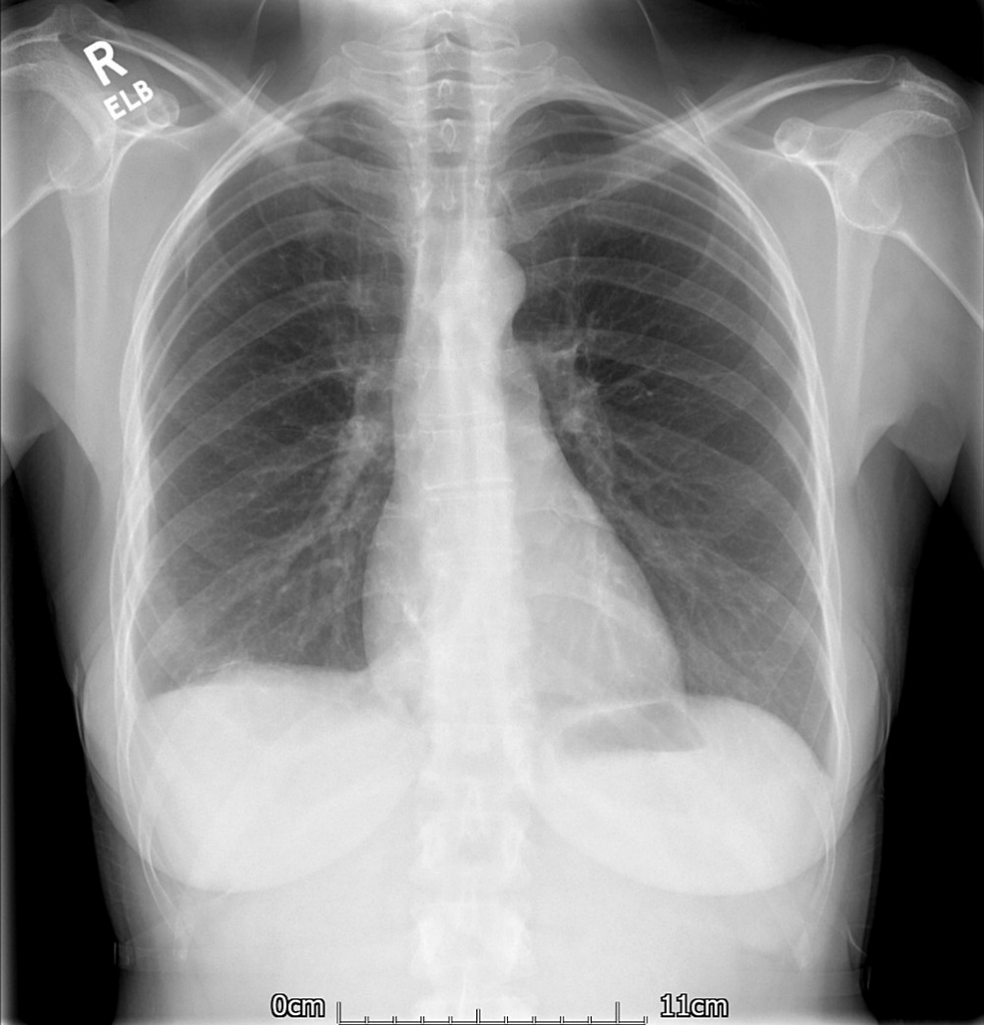
Posteroanterior (PA) chest X-ray, performed on the sixth post-operative day, demonstrating clear lung field bilaterally. No obvious masses, nodules, consolidation or collapse visible. No mediastinal shift.

The patient was referred to the cardiothoracic surgeons for surgical resection of the tumour. The procedure was performed under general anaesthesia with the use of a double-lumen endotracheal tube (ETT). Surgical access was obtained by performing a right posterolateral thoracotomy through the fifth intercostal space (5^th ^ICS). Upon entering the pleural cavity, a large, encapsulated mass was visualised. It was adherent to the adjacent lung parenchyma but not the parietal pleura. The adhesions were dense and highly vascular, and dissection resulted in considerable oozing with an estimated intra-operative blood loss of 1100 ml. The lower pole of the tumour was attached to the diaphragm via a vascular pedicle. However, once the tumour was mobilised, it was easily resected by applying a vascular stapler across the base of the pedicle. Macroscopically, a well-circumscribed and encapsulated mass was resected. It measured 23.1 cm x 21.0 cm x 11.5 cm and weighed 3640 grams. All specimens obtained were sent for histopathological analysis. The patient made an uneventful recovery and was discharged from the hospital on the fifth post-operative day. A chest X-ray, performed prior to her discharge, showed clear lung fields bilaterally with no visible masses, nodules, consolidation or collapse. Additionally, there was resolution of the pre-operative mediastinal shift (Figure 4).

Histopathology of the tumour specimen confirmed the diagnosis of a benign solitary fibrous tumour of the pleura (SFTP) with an intermediate potential for malignancy. The tumour was composed of uniform stellate and spindle cells, set within a fibrocollagenous matrix, with scattered ectatic, branching blood vessels. The mitotic count was up to 1/10 high-power fields (HPF) and there was no tumour necrosis. At six months, the follow-up CT scan demonstrated no radiological features suggestive of disease recurrence.

## Discussion

Solitary fibrous tumours of the pleura (SFTP), also called pleural fibromas, are rare mesenchymal cell tumours that account for <5% of all pleural tumours [1]. It is frequently asymptomatic and is diagnosed incidentally on imaging performed to investigate an unrelated disease. The peak incidence of diagnosis is between the age of 60 to 70 years. It affects both men and women equally with no gender preference [2]. It is usually benign, although, even benign SFTP may have intermediate malignant potential. Around 15-20% are malignant. Certain factors that are suggestive of a malignant SFTP include infiltration of surrounding structures (e.g., adjacent lung parenchyma, the chest wall, mediastinum) and the propensity to recur following surgical resection [5]. Surveillance with routine (e.g., 6-monthly) CT scans has been recommended [1,2].

The aetiology of SFTP is unknown. It has no causal relationship with tobacco or asbestos exposure [1,4]. Histologically, SFTP is composed of pericytes and submesothelial fibroblast, and originates from perivascular multipotent mesenchymal cells. They can either be pedunculated or sessile [6]. Approximately 80% of all SFTP arise from the visceral pleura. Notably, those that arise from the parietal pleura have a greater likelihood of malignancy. They usually develop within the mid-to-lower hemithorax [3]. These tumours vary considerably in size, and although, there is no fixed cut-off to define a "giant" pleural fibroma, the existing literature implies that a pleural fibroma measuring >10 cm in any dimension is considered "giant" [2-5].

SFTP are characteristically slow-growing tumours. In the initial stages, when SFTP are small in size, they are asymptomatic, and are commonly detected incidentally owing to the vast number of scans (including chest X-ray and CT scans), that we perform in the modern era of clinical practice. However, in the rare cases where they remain undetected, the tumour grows in size resulting in symptoms attributable to its mass effect, including dyspnoea, persistent cough, atypical chest pain or tightness, and haemoptysis. Large or "giant" tumours can compress mediastinal structures, including the heart and the lungs, which may result in acute heart failure or severe respiratory distress [1-4].

Rarely, SFTP can present with paraneoplastic syndromes, namely hypertrophic pulmonary osteoarthropathy (~35%) and severe symptomatic hypoglycaemia (~5%). However, this is more commonly seen with malignant SFTP. Clinically, hypertrophic pulmonary osteoarthropathy (HPOA) presents with digital clubbing and painful large joints, especially involving the lower limbs. Intermittent episodes of severe hypoglycaemia occur with the secretion of insulin-like growth factor by the tumour itself [1,3].

With the help of imaging, we can better characterise thoracic lesions or masses. It also aids with operative planning, by providing excellent anatomical description of the tumour and its relation to the surrounding structures. In clinical practice, a chest X-ray is usually the first-line imaging that is requested if there is a clinical suspicion for a thoracic pathology. It is a readily available investigative tool, which can quickly eliminate common differentials, such as a pneumonia, pneumothorax, pleural effusion, and bronchogenic carcinoma. SFTP is characteristically seen as a well-circumscribed, round, or lobulated mass, which may be associated with a pleural effusion on a chest X-ray [2]. However, just as in our case, the best imaging modality for tissue characterisation and delineating the anatomy of the tumour is a contrast-enhanced CT scan of the chest. The CT findings in SFTP are non-specific and are usually dependent on its size; smaller tumours are homogenous and well-demarcated. Whereas a large SFTP appears heterogenous with contrast-enhancement, contains tiny calcifications, and areas of necrosis or haemorrhage. Moreover, with larger tumours, even with a CT scan, it may be difficult to comment on whether the tumour involves the parietal pleura [2,7]. Generally, as the size of SFTP increases, the likelihood of malignancy also increases, as does the heterogeneity of the tumour. Nevertheless, CT has no role in differentiating between a benign and malignant SFTP [5].

Magnetic resonance imaging (MRI) or angiography are used for the assessment of tumour infiltration/ characterisation and in identifying feeding vessels, respectively. Unlike CT scans, SFTP have characteristic features on an MRI scan. They are isointense on T1-weighted sequences and demonstrate variable intensity on T2-weighted sequences. Furthermore, on a T2-weighted sequence, areas of necrosis or haemorrhage appear as regions of high signal intensity [2]. Given the non-specific appearance of SFTP on CT scan, a definitive diagnosis requires histopathological and immunohistochemical analyses.

Approximately 15% of SFTP are identified as malignant on the basis of a combination of radiological, histopathological and immunohistochemical factors [5]. In terms of imaging, a tumour that measures >10 cm in any dimension, demonstrates interval progression in size, infiltrates the chest wall, contains areas of necrosis/ haemorrhage, and/or has an associated pleural effusion, has a greater probability of being malignant. Histological findings consistent with malignancy include hypercellularity, pleomorphism, and a mitotic index of >4/10 HPF [2,8].

Complete surgical resection of the tumour is the only treatment with curative potential. However, a conservative approach with watchful waiting may be adopted for smaller, asymptomatic tumours. Recurrence is rare with approximately 2% for pedunculated and 8% for sessile tumours. Surveillance with serial CT scans at 6-monthly intervals is recommended [1,2,4].

## Conclusions

In the case described above, the patient presented with a history of progressively worsening shortness of breath, and a solitary fibrous tumour of the pleura (SFTP) was diagnosed with the aid of imaging and histopathology. SFTP has non-specific features on imaging, however, a definitive diagnosis may be established with histopathology and immunohistochemical analysis. The tumour may be resected surgically, and in our case, surgical access was obtained via a right posterolateral thoracotomy.
